# Decellularisation of human meniscus tissue using sodium dodecyl sulphate (SDS): Preserving biomechanical integrity for scaffold‐based meniscal repair

**DOI:** 10.1002/jeo2.70375

**Published:** 2025-08-27

**Authors:** Dominic Simon, Benjamin Bartz, Manuel Kistler, Susanne Mayer‐Wagner, Peter E. Müller, Thomas R. Niethammer, Boris M. Holzapfel, Gautier Beckers

**Affiliations:** ^1^ Department of Orthopaedics and Trauma Surgery Musculoskeletal University Center Munich (MUM), University Hospital, LMU Munich Munich Germany; ^2^ Experimental Orthopaedics University Hospital Jena, Waldkliniken Eisenberg Eisenberg Germany

**Keywords:** compressive biomechanical properties, decellularization, knee, meniscus tissue engineering, scaffolds

## Abstract

**Purpose:**

Meniscal tears are common knee injuries and a major risk factor for secondary osteoarthritis. Recently, there has been a paradigm shift toward meniscal preservation, reflecting the meniscus's vital role. In this context, tissue engineering approaches such as the development of meniscal scaffolds have gained attention. However, to reduce the immune response and improve biocompatibility, decellularization of allografts, while preserving the histoarchitectural and meniscal properties, is essential. The current study aimed to evaluate the effectiveness of decellularization and its impact on the biomechanical properties of the human meniscus.

**Methods:**

Twenty‐one human meniscus specimens were collected between July and December 2023 during total knee arthroplasty. Preoperative MRI was performed to verify meniscal integrity. The specimens were decellularized using a sodium dodecyl sulphate (SDS) protocol and compared to native meniscus samples in terms of cell count, assessed through hematoxylin and eosin staining, and biomechanical properties, specifically Young's modulus, measured using a universal testing machine (Zwick Z010).

**Results:**

The cell count in the decellularized menisci was 11 cells/mm² (SD = 13; 95% CI: 2–20), representing a significant reduction compared to the native meniscus samples, which had a cell count of 111 cells/mm² (SD = 42; 95% CI: 81–141; *p* < 0.01). Young's modulus of elasticity was 35.3 versus 36.8 MPa in the anterior region (*p* = 0.8), 32.6 versus 35.6 MPa in the central region (*p* = 0.7) and 36.5 versus 35.8 MPa in the posterior region (*p* = 0.9) for native versus decellularized samples, respectively.

**Conclusions:**

This study demonstrated that the modified SDS‐based decellularization protocol effectively decellularizes the human meniscus. Moreover, the decellularized tissue retained biomechanical properties comparable to those of native meniscus tissue. Tissue decellularization is a promising technique in regenerative medicine, enabling the use of scaffolds for tissue repair, particularly in applications such as meniscus transplantation following meniscectomy.

**Level of Evidence:**

Level III, controlled laboratory study.

**Abbreviations**
3 d: three dimensional
BMSCs: bone marrow mesenchymal stem cells
C: celcius
CI: confidence interval
Decell: decellularized
DNA: deoxyribonucleic acid
DNAse: deoxyribonuclease
ECM: extracellular matrix
GAG: glycosaminoglycan
h: hour
H&E: hematoxylin and eosin
Max: maximal
Min: minimal
mm: milimeter
MPa: megapascal
MRI: magnetic resonance imaging
N: newton
OA: osteoarthritis
PBS: phosphate‐buffered‐saline
pH: pH‐value
RNAse: ribonuclease
SD: standard deviation
SDS: sodium dodecyl sulphate
SOP: standard Operating Procedure
TKA: total knee arthroplasty
μCT: micro‐computed tomography

## INTRODUCTION

Meniscal tears are a common cause of consultation in orthopaedic surgery and often require surgical intervention when symptomatic [4, 20, 28]. In most cases, partial meniscectomy remains the only viable treatment option, as lesions frequently occur in the avascular white‐white zone, which has limited self‐healing capacity [5]. However, removal of meniscal tissue compromises its essential functions—such as load transmission, joint stabilisation and shock absorption—thereby accelerating the onset of secondary osteoarthritis (OA) [10, 12, 13, 23].

In this context, tissue engineering approaches such as the development of meniscus scaffolds, have gained significant attention as a means of preserving meniscal function. For these scaffolds to effectively prevent secondary OA in the knee joint, they must closely replicate the biomechanical properties of native meniscus tissue such as joint's stabilisation and shock absorption functions while supporting the ingrowth of donor cells [2, 6, 17, 18, 19].

Meniscus scaffolds, whether biological or synthetic, are often pre‐populated with mesenchymal stem cells in vitro, as this has been shown to significantly enhance tissue regeneration compared to acellular scaffolds [18]. Different scaffolds provide a matrix for cell adhesion and proliferation. Synthetic scaffolds, such as those made from polymers like polyurethane, are often considered inferior to biological scaffolds, which include acellular meniscus tissue and collagen‐based scaffolds [24]. One key advantage of acellular meniscus tissue over synthetic scaffolds is that it does not require resorption, potentially offering long‐term biomechanical benefits [8, 19].

Currently, three scaffolds are in use: the Collagen Type I Meniscal Implant (CMI) (Stryker, Greenwood Village, CO 80111), the Actifit synthetic polymer meniscal scaffold (Orteq Sports Medicine Ltd., London, SW19 1QT, UK) and the Nusurface artificial meniscal implant (Active Implants, Memphis, TN 38119) [6]. Despite their use, these scaffolds still face several challenges, including suboptimal mechanical properties, the risk of inflammatory responses, biological disintegration, foreign body wear, synovitis, and variable long‐term durability.

Human allogenic decellularized meniscus tissue presents a promising alternative to xenogenic tissue, as it better preserves the native meniscus properties, including its unique microstructure necessary for natural cell attachment and integration [1]. However, the effect of the decellularization process on the biomechanical properties of the meniscus requires further analyses.

Allograft implantation has become a promising strategy for meniscus repair, especially when autologous tissue is unavailable or unsuitable. To reduce immune response and improve biocompatibility, decellularization of allografts is essential. Techniques like chemical treatments (sodium dodecyl sulphate [SDS], Triton X‐100), enzymatic digestion, and physical methods (freeze‐thaw cycles) are commonly used to remove cellular material while preserving the extracellular matrix (ECM). Each method has its advantages and disadvantages: SDS effectively removes cells but can compromise ECM integrity, while Triton X‐100 is milder but may leave residual cells. Recent studies show that combining techniques enhances decellularization efficiency while maintaining tissue architecture. These decellularized scaffolds can be used directly or as templates for recellularization. Additionally, synthetic materials like polycaprolactone, polylactic acid, and polyurethane, as well as hybrid scaffolds combining biological and synthetic components, offer improved mechanical strength and bioactivity [8, 11].

Despite the use of various scaffolds and cell types, fully replicating the complex properties of the native meniscus remains challenging. The current study aimed to evaluate the effectiveness of decellularization and its effects on the biomechanical properties of the human meniscus. We hypothesise that SDS decellularization preserves the native biomechanical properties of human meniscus tissue.

## MATERIALS AND METHODS

Intact medial and lateral human meniscus specimens were prospectively obtained during total knee arthroplasty (TKA) procedures between July and December 2023. Inclusion criteria comprised all patients between 18 and 80 years of age undergoing primary TKA at our institution. To verify meniscal integrity, all patients underwent MRI within three months prior to surgery. Patients were excluded if they had no preoperative MRI, MRI‐detected meniscal tears or defects, a prior history of knee surgery, or macroscopically visible meniscal damage during specimen collection. A total of 21 meniscus specimens, 11 medial and 10 lateral, were collected from 13 patients, two medial and three lateral menisci were excluded due to meniscal tears. The mean age of the donors was 58 years (SD = 12; 95% CI: 49–66; range: 40–80).

Ethical approval for this study was obtained from our institutional review board. As the specimens were anonymized and would otherwise have been disposed of, informed consent was not required. (Ethikkommission der Medizinischen Fakultät der Ludwig‐Maximilians‐Universität, reference number 18‐687 UE).

**Figure 1:**
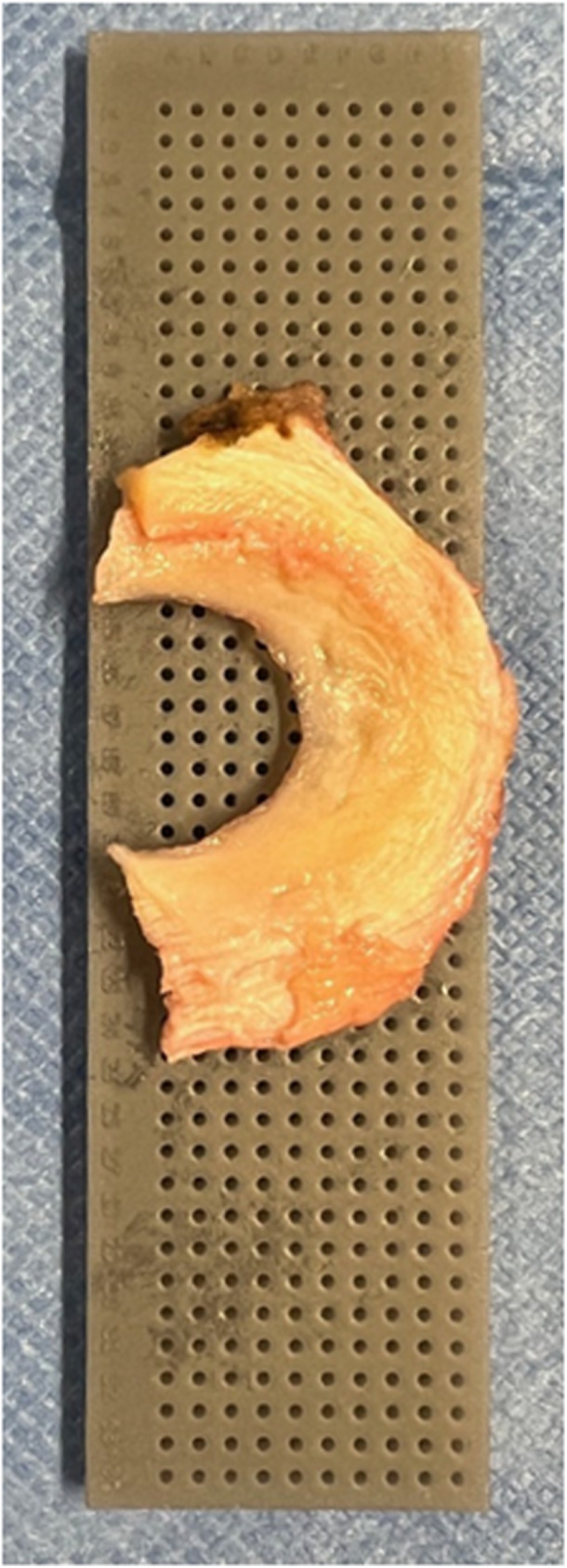
Standardised dissected human meniscus specimen showing a medial meniscus sample on a 3D printed carrier.

All menisci were carefully trimmed with a scalpel to remove excess soft tissue and subsequently placed onto 3D‐printed specimen slides for standardised processing (Figure 1).

Furthermore, small samples of the native meniscus, which were subsequently decellularized, were cut and collected. All samples were freshly frozen at –20 °C. A small representative subset of menisci was scanned using live‐dead assays prior to collection to ensure the viability of the tissue.

Although studies have confirmed that a low number of freeze–thaw cycles (<3) do not alter the macroscopic or biomechanical properties of tissue [14, 16], we conducted tests comparing the biomechanical properties of freshly frozen human meniscus samples with fresh, non‐frozen samples and observed no significant differences. Furthermore, both native and decellularized meniscus samples underwent a single freeze–thaw cycle: they were fresh‐frozen immediately after harvesting, thawed for native testing, and then subjected to decellularization and subsequent analysis.

For decellularization, the samples were treated as follows: native human meniscus was incubated in deionized water for 24 h, followed by 2% SDS for 10 days. In the next step the tissue was washed sequentially in deionized water, 70% ethanol for 24 h, and phosphate‐buffered‐saline (PBS) [14].

### Staining

The native and treated menisci were sectioned and embedded in paraffin in a standardised manner as follows:

Tissue preparation: After thawing, tissue was fixed in 4% buffered formalin overnight. (approximately 20 h) (rate of formalin permeation 1 mm²/h). After that, the tissue was washed with sterile water and then rinsed three times with PBS. Tissue dehydration was performed using the automated and standardised STP‐120 Rotational Tissue Processor from Thomas Medical (5751 Mitterhofen, Austria). The next morning, the samples were casted into paraffin blocks using the Histocore Arcadia H + C paraffin embedding station (Leica Biosystems, 35578 Wetzlar, Germany). The preparations were solidified on an ice plate to form solid paraffin blocks. The paraffin blocks were stored in a refrigerator at 4°C. Cutting of the paraffin blocks was performed using a microtome and histological staining sections were prepared following a standard operating procedures (SOP) protocol for the paraffin embedding station. Hematoxylin‐eosin staining (HE): Samples were dewaxed in 100% xylene, then rehydrated via a descending ethanol series, rinsed with distilled water and then stained by Hemalaun for 12 min. After washing in clean tap water (10 min) (alkaline, pH > 7), followed by Eosin, in aqueous 0.1% solution for 1 min, shortly rinsed with distilled water and then dehydrated in an ascending ethanol series, followed by Xylene, mounting medium for xylene samples and cover slip. The locations of the sections were determined based on overall anatomy by direct visualisation.

### Live dead assays

The live dead assay was used to distinguish between live and dead cells, ensuring the vitality of meniscus tissue at the time of sample harvesting. Briefly, fresh meniscus tissue samples were incubated in a staining solution containing calcein‐AM and ethidium homodimer. After a 30‐min incubation, the samples were analysed using fluorescence microscopy.

### Biomechanical testing

All tests of the biomechanical properties were performed following the standard SOP for testing human meniscus samples in our laboratory. Compression tests to determine the Young's modulus were performed using the universal testing machine ‘Zwick Z010’ (ZwickRoell, Ulm, Germany) (Supporting Information: Figure 1). The Young's modulus values of the menisci were determined using a custom MATLAB code specifically developed for this purpose. These values represent the stiffness of the meniscal tissue and are crucial for understanding its mechanical response under load.

These tests were conducted on the menisci in the ‘anterior’, ‘central’ and ‘posterior’ regions, with each spot tested twice. The tip of the testing head was an aluminium cylinder with a diameter of 2 mm. To secure the menisci during testing, a perforated grid plate was custom‐manufactured. The menisci were fixed onto the grid using surgical sutures. The additive manufacturing was performed using the ‘Form 3+’ printer (Formlabs, Somerville, United States) and ‘Grey V4’ resin (Formlabs, Somerville, USA).

During the tests, the testing machine operated at a speed of 5 mm/min until a preload of 0.1 N was reached. Once this force was reached, the testing speed was increased to 20 mm/min until a 30% compression of the menisci was achieved. All samples were stored in a NaCl solution at all times, except during mechanical testing, which was conducted without additional fluid immersion. Prior to testing, the surface of each meniscus sample was carefully prepared by making a fine incision to create a flat and even surface, ensuring optimal contact with the testing apparatus and consistent loading conditions.

To determine the thickness of the menisci prior to testing, imaging techniques were used. This included checking for calcified areas and identifying suitable testing spots. Based on the analysis of micro‐computed tomography (μ‐CT) images, suitable testing locations within the meniscal tissue were identified. These selected regions showed no signs of calcification, which appeared in the μ‐CT scans as brighter areas within the meniscus, as described by Hellberg et al. [15]. Furthermore, regions exhibiting structural defects or perforations, visible as dark areas in the scans, were excluded from consideration. Only areas with relatively flat surfaces were chosen to ensure uniform contact during mechanical testing and to maintain the reliability of the measurements. The evaluation and selection of appropriate regions were performed by a research associate with prior experience in radiological analysis gained through previous projects.

For the computed tomography imaging, a μCT device (CT‐Alpha, ProCon X‐Ray GmbH, Sarstedt, Germany) equipped with a nanofocus X‐ray source (XWT‐225‐TCHE, X‐Ray‐Worx, Garbsen, Germany) was utilised. The CT data were analysed using the software ‘X‐Aid’ (Mitos GmbH, Germany) and ‘Dragonfly’ (Glassbox Technologies, Los Angeles, United States).

### Statistical analysis

The Young's modulus was determined using a MATLAB code (R2023a, MathWorks, Natick, United States), and the statistical analysis was conducted with ‘IBM SPSS Statistics’ (Version 29.0.2.0, IBM, Armonk, United States). To assess the normality of the data, the Kolmogorov–Smirnov test and the Shapiro–Wilk test were applied, along with a visual inspection using Q–Q plots. To evaluate statistically significant differences between paired groups, the Wilcoxon signed‐rank test was used.

For statistical analysis the Student's t‐test was used. A *p*‐value of < 0.05 was considered statistically significant (*p < 0.05, **p < 0.01).

All statistical analyses were run in GraphPad Prism 10.1.2 (GraphPad Software LLC, Boston, Massachusetts USA) and R Statistical Software (Version 2024.04.2 for macOS, R Core Team 2022, Vienna, Austria).

## RESULTS

### Anatomy

**Figure 2:**
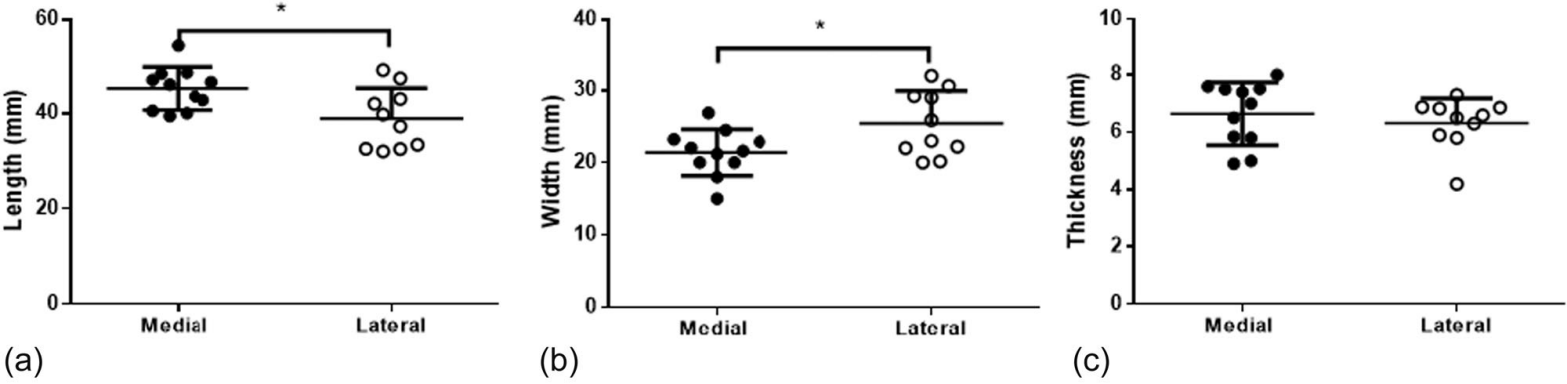
Anatomy of medial and lateral meniscus samples before freezing with comparison of length (a), width (b) and thickness (c) in mm.

The freshly harvested 11 medial meniscus samples measured approximately 45.2 mm (SD = 4.5, 95% CI: 42.2–48.3) in length and 21.4 mm (SD = 3.2, 95% CI: 19.2–23.5) in width. The thickness of the medial meniscus was 6.6 mm (SD = 1.1, 95% CI: 5.9–7.4). In comparison the 10 lateral meniscus samples were significantly shorter, measuring 38.9 mm in length (SD = 6.3, 95% CI: 34.4–43.5, *p* = 0.04) but significantly wider, with a width of 25.4 mm (SD = 4.5, 95% CI: 22.2–28.6, *p* = 0.03). The thickness of the lateral meniscus samples was 6.3 mm (SD = 0.9, 95% CI: 5.7–6.9), showing no significant difference to the medial samples (Figure 2).

### Live dead assays

**Figure 3:**
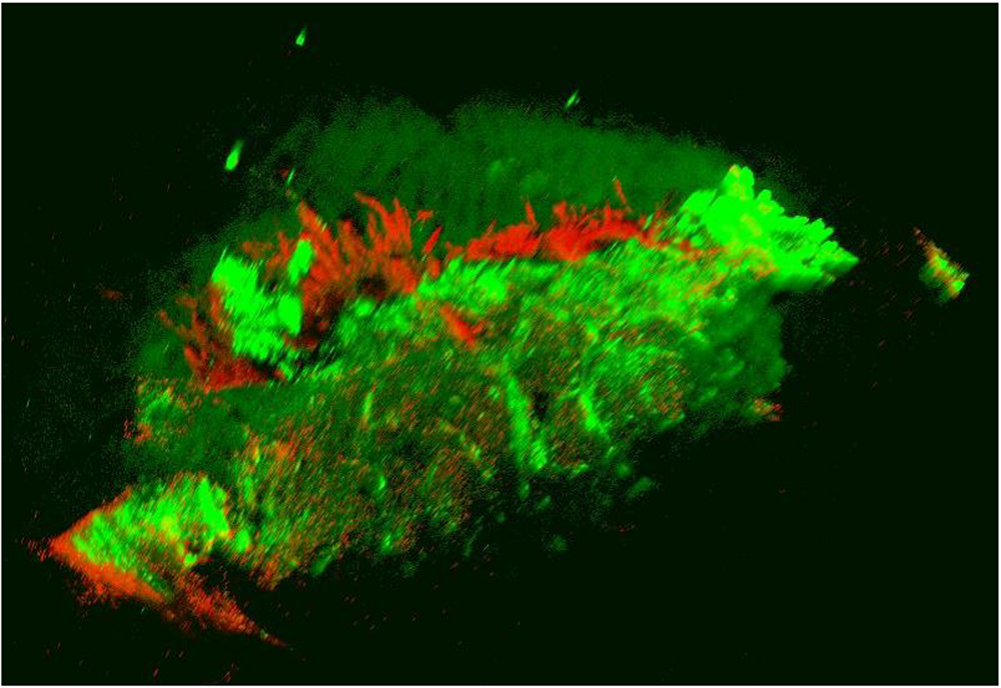
Dead‐live assays of medial meniscus samples validating vital cells (green) after sample collection. The green fluorescent Calcein‐AM shows intracellular esterase activity in the central meniscal regions, thus confirming the presence of vital cells. In contrast red fluorescent Ethidium‐Homodimer‐1, representing avital connective tissue, was shown in some outer soft tissue.

To confirm the vitality of meniscal tissue at the time of harvest, live dead assays were performed following the manufacturer's instructions (Figure 3).

### Cell count

The cell count in the central sections of the menisci was 11 cells/mm² (SD = 13; 95% CI: 2–20) in the decellularized samples, representing a significant reduction compared to the native meniscus samples, which had 111 cells/mm² (SD = 42; 95% CI: 81–141; *p* < 0.01).

**Figure 4:**
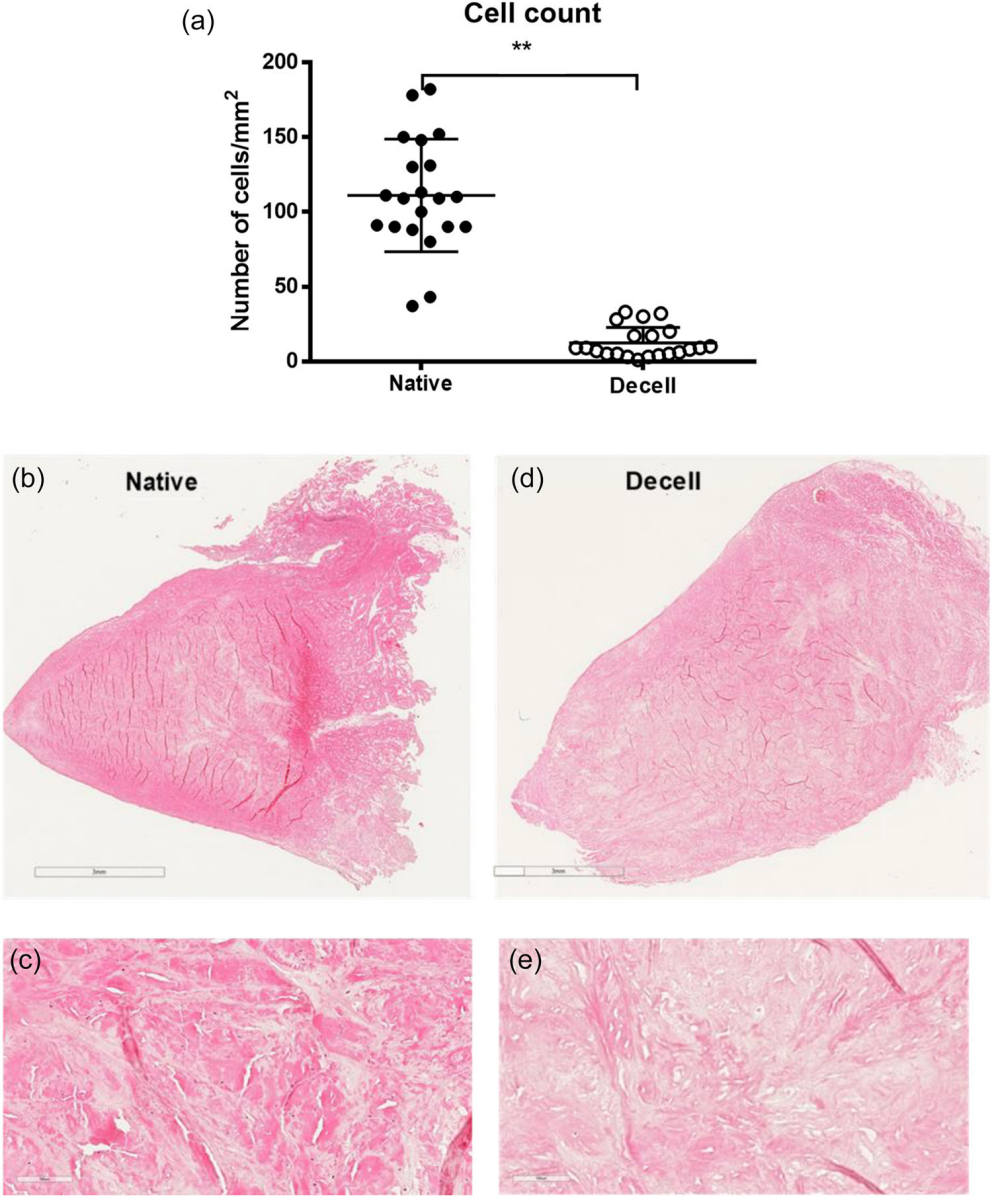
(a) Number of cells/mm^2^ in native and decellularised human meniscus. (b) Hematoxylin and eosin (H&E) staining of a native meniscal section of a medial right meniscus with (c) the nuclei stained blue, while the extracellular matrix and cytoplasm appeared pink, allowing for the quantification of cells within the tissue sections. (d) and (e) Show decellularized sections of a medial right meniscus.

Representative photomicrographic images of a native meniscal section and a decellularized meniscal section of a medial right meniscus are shown in Figure 4.

### Biomechanical testing

The results are presented in Table 1, with data organised according to the three anatomical regions of the meniscus: anterior (front section), central (middle section), and posterior (back section). Each region is further subdivided into two groups: native, representing untreated meniscal tissue, and decellularized, referring to tissue that has undergone the decellularization process.

**Table 1:** Determined Young's modulus values in MPa categorised into ‘anterior native’, ‘central native’, ‘posterior native’, ‘anterior decellularized’, ‘central decellularized’ and ‘posterior decellularized’.

Young's modulus	Native	Decellularized	*p* value
	MPa (SD, 95% CI, range)	MPa (SD, 95% CI, range)	
Anterior	35.3 (22.8, 24.7–46, range 6.7–91.1)	36.8 (21.5, 26.7–46.9, range 5.8–75.4)	0.8
Central	32.6 (21.1, 22.2–43.1, range 4–77.5)	35.6 (22.3, 24.6–47.7, range 2.4–89.5)	0.7
Posterior	36.5 (21.5, 26.7–46.3, range 2.8–83)	35.8 (20.4, 26.5–45.1, range 12.7–79.5)	0.9
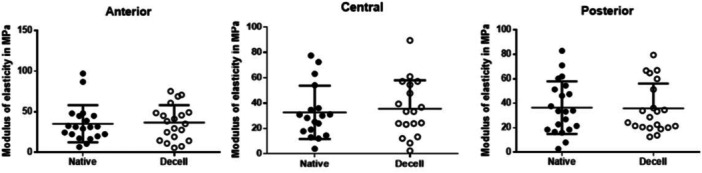			

Abbreviations: CI, confidence interval; Decell, decellularized; Max, maximal; Min, minimal; MPa, megapascal; SD, standard deviation.

Statistical analysis was conducted to compare the native and decellularized groups across all meniscal regions. Although there were variations in mean modulus of elasticity values, all *p*‐values were greater than 0.05, indicating no statistically significant differences in tissue stiffness between the native and decellularized samples.

The mean modulus of elasticity was 35.3 versus 36.8 MPa in the anterior region (*p* = 0.8), 32.6 versus 35.6 MPa in the central region (*p* = 0.7) and 36.5 versus 35.8 MPa in the posterior region (*p* = 0.9) for native versus decellularized samples, respectively.

Further information, including standard deviations, confidence intervals, and ranges, are presented in Table 1.

## DISCUSSION

The primary finding of this study is that the modified SDS based decellularization protocol enables effective decellularization of the meniscus while preserving its biomechanical properties, which are essential for normal knee joint function [1]. We furthermore applied a specialised decellularization protocol to human meniscus samples, which successfully reduced cellular content, thereby minimising the risk of immune rejection and potential inflammatory responses following implantation. The effectiveness of the decellularization process was confirmed by histological analysis, demonstrating a significant reduction in cellular material from 111 cells/mm² to 11 cells/mm² (*p* < 0.01), while preserving the overall histoarchitecture of the meniscus. Compressive modulus measurements further indicated that the decellularized tissue retained biomechanical properties, strength and elasticity, comparable to those of native meniscus tissue, as detailed in Table 1. Moreover, this modified protocol for human meniscus, with a total duration of 12 days, was approximately 15% shorter than the original protocol, offering the advantage of reduced processing time [26].

Several decellularization protocols have been investigated to date, particularly those utilising SDS and Triton X‐100. More recently, these detergents have been used in combination with enzymatic treatments such as deoxyribonuclease and ribonuclease to enhance the removal of cellular components and nucleic acids while minimising ECM degradation [9].

Preserving the ECM, the collagen architecture and glycosaminoglycan content, through strategies such as crosslinking, supplementation or hydration with hyaluronic acid or platelet‐rich plasma may help ensure that the scaffold can effectively withstand physiological loads and stresses after implantation [7, 21, 29].

The successful combination of effective decellularization with preserved biomechanical properties presents a promising strategy for meniscal repair. The decellularized meniscus scaffold offers an optimal environment for host cell infiltration and tissue remodelling. Additionally, its biomechanical robustness ensures that the scaffold can function effectively within the joint, supporting normal knee mechanics and potentially enhancing the overall success of meniscal repair procedures [27].

To achieve this, scaffolds should ideally possess intrinsic biomechanical properties comparable to native meniscus tissue, ensuring its role as a stabiliser and shock absorber, while also promoting donor cell ingrowth. Human allogenic decellularized meniscus tissue offers a promising solution compared to xenogenic alternatives, as it more closely mimics the native meniscus, particularly its unique internal microstructure, thereby supporting natural cell attachment and integration [1].

Human mesenchymal stem cells have been used to repopulate decellularized scaffolds. Furhermore bioreactor systems that simulate physiological loading through mechanical stimulation have been shown to enhance ECM deposition and recellularization. Moreover, in vivo studies in animal models have demonstrated the biocompatibility and partial integration of decellularized meniscal scaffolds [3].

The scaffolds provide a biocompatible and structurally suitable matrix that supports cellular infiltration and tissue regeneration, as recently demonstrated by He et al. in a porcine meniscus model. Their study showed a significant reduction in ECM DNA content and preservation of ECM integrity following decellularization with Triton X‐100 and SDS, with no residual nuclei observed [14]. Furthermore, biomechanical assays indicated the retention of collagen, and glycosaminoglycan content. Moreover, the scaffolds also demonstrated biocompatibility, as bone marrow mesenchymal stem cells successfully proliferated over a 2‐week period in vitro.

This study has several limitations. First, the relatively small sample size and the anatomical heterogeneity between medial and lateral meniscus samples. However, it is one of the studies with the largest number of meniscus specimens compared to existing literature [22, 26].

Additionally, the samples were obtained from patients with OA. While structural integrity was ensured through MRI scans, and macroscopically degenerative menisci were excluded during harvesting, some degree of degeneration cannot be fully ruled out. Despite these limitations, the use of human specimens offers the advantage of directly evaluating the decellularization process and biomechanical properties in a human‐specific context, as opposed to animal models, which may not fully replicate the complexities of human meniscus. Lastly, it is possible that in degenerated menisci, decellularization may have a limited biomechanical effect, further emphasising the importance of the extracellular matrix itself.

Future research should focus on optimising decellularization protocols to achieve a better balance between complete cell removal and ECM preservation, as incomplete decellularization remains a challenge—particularly in the dense central regions of the meniscus. Furthermore, bioprinting is gaining interest as a method to create customised, patient‐specific scaffolds [25]. Even if promising, the clinical translation of decellularized meniscus scaffolds remains in its early stages. Robust in vivo studies are needed to assess their long‐term biomechanical performance, recellularization potential, and integration into host tissue. In parallel, incorporating bioactive molecules or growth factors into these scaffolds may further enhance cellular infiltration and tissue regeneration, potentially leading to more effective and biologically active meniscal repair solutions.

## CONCLUSION

This study demonstrated that the modified SDS‐based decellularization protocol with reduced processing time effectively decellularizes the human meniscus while preserving its overall histoarchitecture. Moreover, the decellularized tissue retained biomechanical properties, such as strength and elasticity, comparable to those of native meniscus tissue. The success of such decellularized scaffolds in meniscal repair could enhance treatment strategies by providing a more durable and biocompatible solution for patients with meniscal injuries, thereby preventing secondary osteoarthritis of the knee and delaying the need for TKA.

## AUTHOR CONTRIBUTIONS

All authors contributed to the design of the study, evaluation of data and writing the manuscript. All authors have released the manuscript for publication. Study conception and design: Dominic Simon. Material preparation, data collection and analysis: Dominic Simon, Benjamin Bartz and Manuel Kistler. First draft of the manuscript: Dominic Simon, Benjamin Bartz and Gautier Beckers. Review of the manuscript: Dominic Simon, Benjamin Bartz, Manuel Kistler, Susanne Mayer‐Wagner, Thomas R. Niethammer, Peter E. Müller, Boris M. Holzapfel and Gautier Beckers.

## CONFLICT OF INTEREST STATEMENT

The authors declare no conflicts of interest.

## ETHICS STATEMENT

Ethical approval for this study was obtained from our institutional review board. As the specimens were anonymized and would otherwise have been disposed of, informed consent was not required. Ethikkommission der Medizinischen Fakultät der Ludwig‐Maximilians‐Universität, reference number 18‐687 UE.

## Supporting information

Supporting information.

## Data Availability

The data that support the fndings of this study are available from the corresponding author upon reasonable request.
