# Supplementary figures and images for: Decellularisation of human meniscus tissue using sodium dodecyl sulphate (SDS): Preserving biomechanical integrity for scaffold‐based meniscal repair
Source: J Exp Orthop. 2025 Aug 27;12(3):e70375. doi: 10.1002/jeo2.70375 (PMC12381530; doi:10.1002/jeo2.70375)

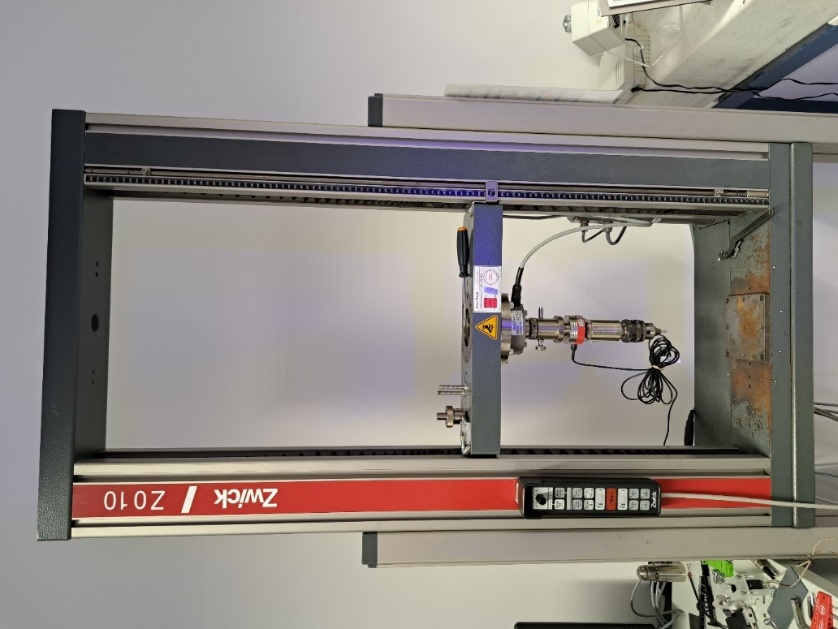

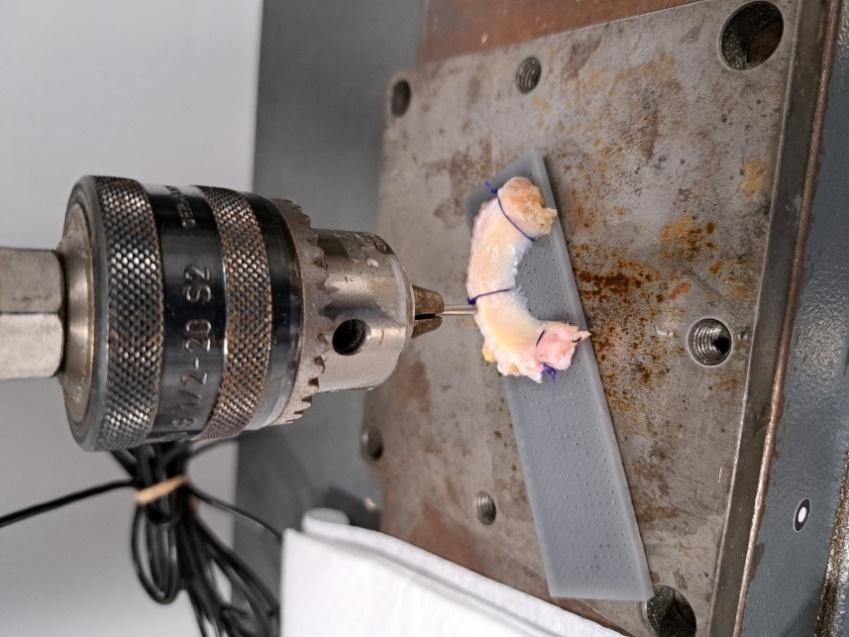


Supplementary Figure 1: Biomechanical testing set up in the universal "Zwick Z010" testing machine.

Supplement: Supplementary file 1 — Supporting information. [file JEO2-12-e70375-s001.docx]
